# Antimicrobial efficacy of *Mentha piperata*-derived biogenic zinc oxide nanoparticles against UTI-resistant pathogens

**DOI:** 10.1038/s41598-023-41502-w

**Published:** 2023-09-11

**Authors:** Nisar Ahmad, Shujat Ali, Muhammad Abbas, Hina Fazal, Saddam Saqib, Ahmad Ali, Zahid Ullah, Shah Zaman, Laraib Sawati, Ahmad Zada, Sohail

**Affiliations:** 1 Center for Biotechnology and Microbiology, University of Swat, Swat, 19200 Pakistan; 2 Pakistan Council of Scientific and Industrial Research (PCSIR) Laboratories Complex, Peshawar, 25120 Pakistan; 3 State Key Laboratory of Systematic and Evolutionary Biology, Chinese Academy of Sciences, Beijing, China; 4 Centre of Plant Science and Biodiversity, University of Swat, Charbagh, Swat, 19200 Pakistan; 5 Department of Botany, University of Malakand, Chakdara, 18800 KPK Pakistan; 6 Department of Chemical and Life Sciences, Qurtuba University of Science and Information Technology, Peshawar, 25124 Pakistan; 7 Institute of Biology/Plant Physiology, Humboldt-University Zü Berlin, 10115 Berlin, Germany; 8 College of Bioscience and Biotechnology, Yangzhou University, Yangzhou, 225009 China

**Keywords:** Microbiology, Plant sciences

## Abstract

Misuse of antibiotics leads to the worldwide spread of antibiotic resistance, which motivates scientists to create new antibiotics. The recurring UTI due to antibiotics-resistant microorganism’s challenges scientists globally. The biogenic nanoparticles have the potential to meet the escalating requirements of novel antimicrobial agents. The green synthesis of nanoparticles (NPs) gained more attention due to their reliable applications against resistant microbes. The current study evaluates the biogenic ZnO NPs of *Mentha piperata* extract against resistant pathogens of urinary tract infections by agar well diffusion assay. The biogenic ZnO NPs revealed comparatively maximum inhibition in comparison to synthetic antibiotics against two bacterial strains (*Proteus mirabilis*, *Pseudomonas aeruginosa*) and a fungal strain (*Candida albicans*).The synthesized biogenic ZnO NPs alone revealed maximum activities than the combination of plant extract (PE) and ZnO NPs, and PE alone. The physiochemical features of ZnO NPs characterized through UV–Vis spectroscopy, FTIR, XRD, SEM, and EDX. The UV–Vis spectroscopy revealed 281.85 nm wavelengths; the XRD pattern revealed the crystalline structure of ZnO NPs. The FTIR analysis revealed the presence of carboxylic and nitro groups, which could be attributed to plant extract. SEM analysis revealed spherical hollow symmetry due to electrostatic forces. The analysis via EDX confirmed the presence of Zn and oxygen in the sample. The physiochemical features of synthesized ZnO NPs provide pivotal information such as quality and effectiveness. The current study revealed excellent dose-dependent antimicrobial activity against the pathogenic isolates from UTI-resistant patients. The higher concentration of ZnONPs interacts with the cell membrane which triggers oxidative burst. They may bind with the enzymes and proteins and brings epigenetic alteration which leads to membrane disruption or cell death.

## Introduction

The clinical utilization of antibiotics such as; penicillin was presented in 1930 and sulfonamide in 1940 during World war-II in 1940. As a result, individuals of that era showed that infectious diseases were completely controlled by antibiotics. But recently, the uncontrolled use of antibiotics develops antibiotic resistance worldwide and nowadays resistance to antibiotics is a crucial public health problem^1^. The worldwide emergence of pathogenes of multi-resistant nature is due to the misuse and overuse of antibiotics in human, veterinary, and agricultural medicines around the world.Antmicrobial resistance (AMR) was noticed shortly afterdiscovery of antibiotics^2^. For instance, resistance to penicillin after its clinical debut in 1942 was noticed in 1947 by *Staphylococcus* strains^3^, The resistance case of streptomycin debuted in 1944 and vancomycin took 30 years after their clinical debut^4^. The debut history of vancomycin starts 65 years ago and remains standard against the infections of methicillin-resistant Staphylococcus aureus. Its long-time resistance is due to controlled utilization of an initial 25 years; therefore, the long delay creates a big opportunity to develop effective antibiotics during the era of antibiotics from the 1950s to the 1960s. Unfortunately, the rise of complicated MRSA makes vancomycin retire practically at 65 years of age^5^. Due to the uncontrolled use of vancomycin across the world vancomycin-resistance *Enterococci* (VRE) appeared between the 1980s and 1990s. It developed in humans due to avoparcin use in livestock where it passed the resistant bacteria from animals to humans^6^. The half way vancomycin resistance strains turned into completely vancomycin-resistance *staphylococcus aureus* reported in 1997 therefore banned by European Union in animal feed^7^.

Approximately in animals 50% of antibiotics used for growth promotion, chemotherapy and prophylaxis that makes the crucial problem^8^. Therefore, many antibiotics are utilized medically and the types of targets prohibited are inadequate. Antibiotics are routinely categorized on the bases of its activity and chemical structure. To understand how microscopic organism to become resistance and how antibiotics work, an epitome description of the main category of antibiotics are needed. The vital categories of antibiotics that inhibit four ideal targets include biosynthesis of cell wall, protein, RNA and DNA, and folate^9^. Either resistance may be innate or acquired. In the condition of innate resistance such that *pseudomonas aeruginosa* relate to alteration in the outer membrane lipopolysaccharide (OM-LPS). In this case, membrane permeability is too much low and a primary cause of innate resistance toward many antibiotics^10^. Acquired resistance is brought about by mutation in chromosome or accession of transposon or plasmid that harbors determined for resistance^7^. In this regard Nanotechnology and Combinatorial chemistry are the major technologies to discover new antibiotics using the combinatorial libraries of polyketides and non-ribosomal peptides^11^. Green synthesis of nanoparticles is eco-friendly, cost-effective, and non-hazardous compared to chemical and physical synthesis methods^12^. Therefore, medicinal plants are also utilized as an antimicrobial agent’ pathogens traditionally. Scientist turned around their focus towards plants to their low side effects and easy availability^13^.

An ethno-medicine report gives us important facts for finding new drugs from medicinal plants. Plants are good way to play important role in human welfare. Medicinal plants are widely used around the world due to free from adverse effects and cheap as compared to antibiotics^14,15^. Instead of success of natural products in drug invention and pharmaceutical, therapeutics companies reduce their achievement in medicinal plant and natural products as a result of difficulties and some drawbacks^16^. The collection of plants and other samples might be difficult and depend upon the conditioned by abundance and accessibility for example for the collection of marine organisms required expansive facilities and expert peoples^17^. Sometimes the natural products or active compounds occurs in small quantities in complex-mixture required a comprehensive isolation and purification process^18^. *Mentha piperata* is a flowering perennial herb and it is grown for large scale uses in food, cosmetics, medicine, and pharmaceutical industries^19^. Among Mentha species, *Mentha piperata* is a fast-growing herb with multifunctional properties. *Mentha piperata* locally called Valayati-Pudina and peppermint, balam mint, Lamb mint, candy mint, and brandy mint generally^20^. *Mentha piperata* is commonly used for the treatment of antispasmodic, as antioxidant, antibacterial, for urinary tract infection, antiallergenic, sedative, and for anti-inflammation^21^. They are economically important for human because they have large scale benefits like pharmaceuticals, fragrance, flavor, dyes, insecticides, and toxins^22^.

Worldwide the oil of *Mentha piperata* is used in chewing gum, candy care, mints chocolate, shampoo, and oral preparation like toothpaste, dental creams, and mouth washer and also displayed the growth inhibition of yeast, fungi and bacteria^23^. The main ingredients are menthol and menthone extracted from fresh parts of flowering plants are well known oil worldwide^24^. In previous time a large number of scientific researchers support the effect of peppermint and its essential oil stimulate the CNS. Peppermint essential oil is used across the world for analgesic effect as they have the capacity to decrease pain and increase flow blood to affected parts^25^. Peppermint oil is also used for skin problem like itching, rashes, acne, bacterial and viral infection. In northern African and Arabian countries, its different parts and formulations (such as; oil, leaf, leaf extract) used for multiple functions and Touarag tea especially used in treating the digestive disorders. In 2050 the population of world will be approximately reached to 9.0 billion which create many problems for the growing population. The production of food rate will be rise approximately 60 to 70 percent^26^. It could be possible to produce good food material only through the use of nonmaterial and the application of nanotechnology in agriculture^27^. Nowadays Bio-nano technology achieved many goals such as development of many devices, materials and techniques which solve large number of human problem and nature like green agriculture, renewable energy production, diagnostic tools for detection of human and plants diseases^28^.

It has been suggested that nanoparticles (NPs) have the ability to in transporting materials across the natural membrane. It can envelope, attach and catch a large variety of both hydrophilic and hydrophobic material and biomaterial like DNAs, RNAs and peptides^29^. Although nano-based medication delivery plat forms make chance to increase stability of medication and bioavailability by minimizing the half-life of delivered nano-bundled medication to the specific organ^30^. A large number of respiratory diseases today are controlled by nano packages such as; M-tb infection, asthma, COPD (Xu, 2022), and lung cancer^31–35^.Among different nanoparticles, the ZnO NPs have less than 100 nm diameters. They have high catalytic activity^36^ and in animal studies these NPs show that due to their small size they can easily travel across the body/membrane and can easily entering to placenta, blood–brain barrier^37^. Zinc oxide nanoparticles have large number of applications such as high piezoelectric property, wide band gap, large binding energy and that is why it is used in optoelectrical and laser devices^38^. Zinc oxide has amazing properties in diagnostic, bimolecular detection, microelectronic^39^. Nanoparticles such as ZnO show different morphologies and exhibits outstanding antibacterial movement over in different range of bacterial species^40^. At present time ZnONPs are considered as antibacterial specialist in both nano-scale and micro-scale formulations. ZnO show outstanding antibacterial properties when the molecule is reduced to nanometer scale and can interface with bacterial surface or potentially enter inside bacterial cell, and later on exhibit bactericidal potential. Some toxic compounds are commonly used to stop the growth of microorganism like in food industry; while ZnONPs are investigated by different researcher as non-toxic for the cell of human. The features such as; the utilization of ZnO NPs as antibacterial agents, toxicity to microorganisms, and control of outstanding biocompatibility of the human cell make it unique^41^.

The different antibacterial properties of nano-materials reduce to their aspect ratio as high specific and display a unique physiochemical effect. Day by day the resistant bacterial infections are major health problem across the globe and a big challenge to social and economic complications. Outbreaks of infectious strains, resistance of antibiotic to bacteria, change in bacterial genome, absence of satisfactory vaccine in progressive nations, and hospital-associated infections are global health threat to population especially in youngster for instance yearly 1.5 million deaths occur from the infections of resistant *Shigella flexneri* while other microbes include *Escherichia coli*, *Clostridium perfringens*, *Pseudomonas aeruginosa, Enterococcus faecalis* and *Salmonella typhi*^42^. ZnONPs is reported as one of the important, functional, strategic, promising and versatile particles for multiple applications including antibacterial properties. In periodic table Zn and O are categorized into two and six group^43^. ZnO shows a light covalent character but exhibits strong ionic bonding. Further, the higher selectivity, longer durability and heat resistance of ZnO are presided than inorganic and organic materials^41^. The synthesis of small sized ZnO is used as new antibacterial molecule due to its specific antifungal and antibacterial applications. ZnO have highest photochemical and catalytic activities. In the UV ZnO have highest optical absorption (325–400 nm) and UVB (280–315) with outstanding antibacterial responses^44^. Nowadays ZnO NPs are used in biomedical and as antiviral agents, due to their potential biocompatibility on the other metal oxides.

Hence, the fundamental aim of this investigation encompassed the formulation of biologically derived Zinc Oxide Nanoparticles (ZnO NPs) sourced from *M. piperata*, primarily intended for employment as antimicrobial agents targeting antibiotic-resistant bacterial strains isolated from human urinary tract infections (UTI). This current study represents a distinctive and pioneering endeavor, marking its novel approach towards assessing the effectiveness of biogenic ZnO NPs in addressing UTI-causing resistant microorganisms, with potential implications for the biomedical industry.

## Materials and methods

### Sample collection and processing

The present study was carried out at Centre for Biotechnology and Microbiology, University of Swat and Pakistan Council for Scientific and Industrial Research (PCSIR), Peshawar. The urine samples were collected from 100 patients at Miangul Abdul Haq Jahanzeb Kidney Hospital [MJKH] (https://www.mjkhkp.gov.pk) formerly known as Nawaz Sharif Kidney Teaching Hospital, Manglawar Swat-Pakistan. The samples were collected with the permission of the administrative authority of the hospital following the ethical and standard procedure as given in^45^. The urine sample from the patients was collected in sterilized containers and stored in refrigerator at 4°Cfor bacterial culture.

### Bacterial and fungal culturing procedure

Nutrient agar (NA) and blood agar (BA) media for culturing was prepared according to the protocol of^46^. A known amount media powdered was poured into boiling water and then autoclaved (121 °C; 20 min; 15 psi) and broad-spectrum antifungal antibiotics were added after autoclaving to inhibit the growth of unwanted fungi in culturing media. The media was allowed for cooling and then transferred to sterile petri dishes and incubated for further processing. Mac Conkey agar (MCA) media was used to isolate gram negative bacterial strains on the basis of lactose fermentation which differentiate bacterial species. The species which ferment lactose display pink color on MCA surrounded by salts precipitation. Due to the production of acid the MCA color changed to red from neutral. The bacteria lacking the ability to ferment lactose appear colorless colonies. The culturing media was placed in incubator (37 °C) for 24 h to check contamination. The urine samples were transfer onto culture media under sterile condition and subsequently the cultured plates were incubated for 24–48 h at 37 °C. After incubation the different colonies appeared on NA was marked using colony morphology. Furthermore, fresh mannitol salt agar media was applied for sub-culturing of marked colonies and again incubated for 24–48 h at 37 °C to isolate pure cultures of bacteria using colony morphology. Furthermore, for the isolation of *C. albicans*, the urine samples were inoculated onto PDA and incubated for 24 h at 37 °C. After inoculation and incubation various fungal colonies appear on PDA. During the procedures, penicillin, ampicillin and ciprofloxacin were added to the culture media to inhibit the growth of unwanted bacteria.

### Identification of pure bacterial and fungal strains

For the identification of pure cultures of *Proteus mirabilis* and *Pseudomonas aeruginosa* different tests were performed including gram staining (GS) and multiple biochemical tests using the procedure of^47^. Pure culture can be identified using the GS from the prepared subcultures. A smear was prepared for each pure culture by excising a small part of the colony using sterilized loop. The smear was placed on glass slide by adding sterile water and then heating it for fixation. Initially the slide was exposed to crystal violet dye for 60 s and then washed with autoclaved H_2_O. Secondly, the slide was exposed to iodine for 60 s and then washed again with autoclaved H_2_O. In the third step the slide containing pure culture was treated with 70% alcohol as decolorizing agent for 60 s and then washed with autoclaved H_2_O. The slide was dried carefully and placed under microscope (40x) using immersion oil. In case of fungal isolation and identification, the fungal colonies were detected through general morphological analysis on PDA media and then the gram staining was also performed and finally the germ tube formation lactophenol staining was done to confirm the target *C. albicans*.

Different biochemical tests were performed for further identification that includes catalase, oxidase, triple sugar iron test, indole test and urease test. For all these tests, the procedure of^48^ was followed. Catalase test was performed to test the catalase enzyme production by the organism. The role of this enzyme is the release of oxygen and water from the H_2_O_2_. If the reaction release bubbles it means that the culture is catalase positive and the specie is gram negative. Coagulase is special test used for the identification of *Pseudomonas aeruginosa*in in which tetra-methyl-p-phenyl diamine di-hydro-chloride reagent was used to identify the oxidase producing bacteria. The positive test revealed that the culture consists of *Pseudomonas* otherwise, negative results represents the presence of family *Enterobacteriaceae*. The TSI test media composed of glucose, sucrose, lactose, phenol red and iron sulphate as indicator. It also displays that the culture is able to release hydrogen sulphide (H_2_S) or not. Pure culture was streak on the surface of slant and stabbed on TSI agar Butt following incubation for 24 h at 37 °C. The tubes were examined in order to measure the change in slant or Butt. If the culture is able to use glucose, then the slant or Butt color changed into yellow due to the production of acid. The appearance of cracks in the media represents the release of bubbles or gas. If no fermentation occurs the slant and Butt color do not change from initial red color. If the culture produces hydrogen sulphide using ferrous sulphate which combined with iron again and form ferrous sulphide and produce black precipitation. Indole test was performed to check the bacterium ability to produce indole from the tryptophan amino acid. In this test the addition of Kovacs reagent changes the media color to cherry red from yellow color. Urease test was also performed in which the enzyme converts urea to ammonia and CO_2_ in the presence of H_2_O. The three components react with each other and produce ammonium carbonate and convert phenol red which is an indicator from orange yellow to bright pink color.

In case of *C. albicans s*lacto-phenol cotton blue staining (LPCB) is a specific technique commonly used for the preparation semi-permanent microscopic fungi. LPCB has three main parts that is phenol used as antiseptic to killed living entities, lactic acid used as preservative agent that preserve and maintain fungal structure and cotton blue strain that provide blue color to fungal cell wall components. Here a drop of 70% ethanol was poured on sterilized glass slide and the specimen was mixed with it, further, one or two drops of LPCB is added to the slide before the ethanol is dried. A cover slip was placed upon the smear carefully to avoid bubble formation. The slide was studied under microscope using higher power of 40X objective lense for detailed investigation of spores and other structures.

### Antibiotics susceptibility test

The disk diffusion method as Kirby-Bauer method of antibiotics was performed for the microbial sensitivity. Through the help of sterilized swab, the targeted microbes were streak uniformly on Mullar Hinton Agar (MHA) plate surface. The cultured plates were kept for 5–6 min for drying and then the antibiotics (Ciprofloxacin, azithromycin and clotrimazole) discs were placed on the surface of the media and these plates were then incubated at 37 °C for 24 h. Here two cultured plates of MHA were used for testing of multiple concentrations. The results were collected as zone of inhibition around the disc and expressed in mm. The obtained results were interpreted as resistance or sensitive or intermediate^49^.

### Preparation of plant extract

Fresh leaves of *Mentha piperata* were utilized for the synthesis of ZnO nanoparticles. The fresh plants were collected from the Medicinal section of the Botanical Garden of PCSIR Peshawar Pakistan. The procedure as adopted in^50^ was followed for extract preparation with minor modifications. Fresh plant leaves of 20 gm were washed with sterile water and shade dried for the formation of dried powder. The powder was mixed with 50 ml distilled water and incubated for 24 h at room temperature. The extract was filtered and centrifuged at 4000 rpm for 40 min. The supernatant was utilized for the synthesis of Zinc Oxide NPs and stored at 5 °C for additional procedure.

### Biosynthesis of zinc oxide nanoparticles

Zinc oxide NPs were synthesized according to the standard procedure^50^. The plant extract consists of different functional groups such as; phenolic, carboxyl, and amine which function to reduce the Zn ions. The plant extract of *Mentha piperata* was mixed with the solution of zinc oxide already taken in a flask. A solution of Zinc dioxide (3.5 mM) was prepared by dissolving Zinc dioxide salt in distilled water to reach a concentration of 0.64218 g/L. The solution was stirred for 4 h at 50 °C using a magnetic stirrer, and the solution was reduced by adding a plant extract drop-wise keeping the PH 7–8 and till the appearance of yellow to red–orange color. The solution was incubated at 30 °C for 24 h. Then the solution was centrifuged at 10,000 rpm for 10–15 min (Centrifuge: Model: Eppendorf 5424 R, Germany, with 24 × 2 ml A-45-24-11 Rotor). After that solution(extract + ZnO NPs) was poured into Petri dishes and dried overnight in the oven to make a powder of nanocrystals to use for additional processing.

### UV – spectroscopy characterization of ZnO Nanoparticles

The UV range of the combined NPs was acquired by scanning of ZnO NPs in between 200 and 600 nm utilizing UV–Vis Halo DB-20 spectrophotometer^51^ and spectrum was captured against specimen with assistance of TU-1901 double–beam U-V Visible spectrophotometer which was dissipated in ethanol.

### FTIR analysis

The confirmation of organic functional group arranged in plant extract and NPs was made with FTIR by potassium bromide (KBr) pellet method with a settlement and examine range of 4 cm^−1^, from 400 to 4000 cm^−1^. The infrared (IR) ingestion spectra uncovered the presence of various functional groups on the particle’s surface.

### XRD characterization of ZnO Nanoparticles

The biogenic synthesized nanoparticles were characterized with different techniques to identify the desired nanoparticles. The ZnO nanoparticles were subjected to XRD. The XRD observations of ZnO NPs were recorded on X-ray diffractometer at the energy range of 20 from 10 to 80 with a scan rate of 20/min utilizing Cu Alpha (lamda = 1.5406A) radiation with an accelerating voltage of 40 kV. Different stages present in the samples were assessed by mean of X-pert high score programming having search match office.

### Scanning electron microscopy (SEM) and Transmission electron microscopy (TEM), Selected Area Electron Diffraction (SAED) and Zeta potential

The structural observations of ZnO NPs were carried out by SEM (model Jeol JSM-6480LV). The SEM was utilized at 20 kV for 4 min. The nanoparticles were settled on stub with the assistance of adhesives, covered with gold. The structural observations of ZnO NPs were also carried out by TEM **(**JEM-2100; manufacturer JEOL, Japan). The TEM was utilized at 200 kV voltage at a magnification of 1,500,000X with a resolving power of 1.4 Å. The charge measured on the surface of the NPs calculated through a test known as Zeta potential (Zetasizer Blue; Malvern Panalytical, UK). As the NPs carry and float a loop of opposite electric charge. So, the NPs surface net charge is screened out using the abundance of ions carrying the opposite charge near the body of NPs. The analysis of SEM, TEM, SAED and Zeta potential observations were carried out in the central resource’s laboratory (CRL), University of Peshawar.

### Energy dispersive X-ray spectroscopy analysis (EDX)

Energy dispersive x-ray (EDX) examination joined with SEM decided the presence of various elements in the NPs sample. The specimen was resolved through EDX programming by applying ZAF correction whereas; Z = atomic number, A = absorbance, and F = fluorescence. The samples data were taken from the ideally shaped particles exposed on the top of the plate to get the last measurement of the element in the sample.

### Antimicrobial activities

In this study two different bacterial strains and one fungal strain were used from the isolated samples of from UTI patients. These bacterial strains include; *Proteus mirabilis* and *Pseudomonas aeruginosa* and fungal strain include; *Candida albicans*. All these microbes are pathogenic and Multi-drug resistant (MDR). Zinc oxide nanoparticle (20 mg) was dissolved in 20 ml distilled water. After that Well diffusion method was utilized for the antimicrobial activity of nanoparticle as described by^52^. The zone of inhibition was measured in mm as diameter around the well. The microbes (*Proteus mirabilis, Pseudomonas aeruginosa* and *Candida albicans*) were streaked in each plate and three well were bored in each plate. After that, each well of the plate was poured with nanoparticle. Exactly, a 100 µL nanoparticle extracts (100 ug/ml) was introduced in first well, then 50 micro liter nanoparticle extracts (25 ug/ml) was introduced into the second well and 25 µL nanoparticle extracts (25 ug/ml) was introduced into the third well to checked the antimicrobial activity of nanoparticle and kept it on 37 °C in incubator for one day. After that Zone of inhibition were recorded in millimeter (mm). For positive control the broad-spectrum antibiotics was utilized.

### Data analysis

The antimicrobial activities of differential treatments were carried in triplicates. For descriptive statistics (mean values ± SE determination) excel sheet was used. For significance and least significant differences of data one-way analysis of variance (ANOVA) Statistics software (v. 8.1; USA) was used. The graphical designing of thee data was carried out by Origin Lab software (v. 8.5; USA).

### Methods/experiments

We confirmed that all methods/experiments were performed following the relevant guidelines and regulations. Further, the protocols used in this study were according to the approved guidelines of the institution.

### Ethics committee approval

All experimental protocols were approved by the ethical committee of Center for Biotechnology and Microbiology, University of Swat, UOS/EC/22-423**.**

### Informed consent

Informed consent was obtained from all patients participating in the study.

### Research involving plants

We confirmed that all methods (field studies/collection) involving *Mentha piperita* (L.) used for the greens synthesis of NPs in the current study were in accordance with relevant guidelines/regulations/legislation.

## Results and discussion

### UV–visible spectrometry

**Figure 1:**
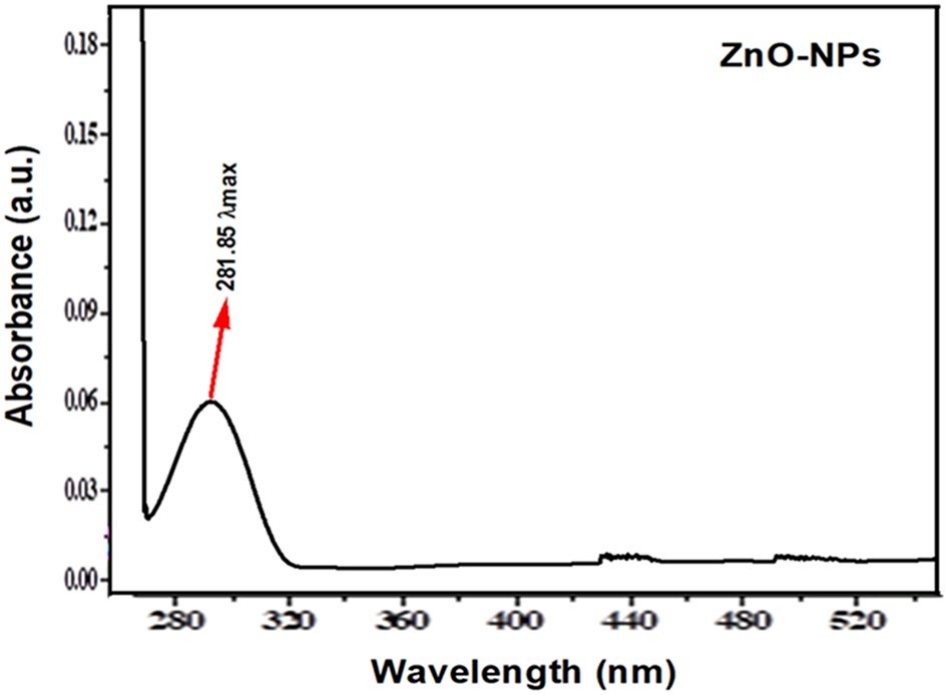
UV- Visible spectrometry of biogenic Zinc NPs prepared from *Mentha piperata.* The antimicrobial ZnO NPs absorption spectra appear in the range of 280 to 320 nm which represent the fine structure of NPs and high band gaps energy arising from strong electronic structures of defects and inner shell electron transitions and finally quantum confinement effect.

In the initial stage, we confirmed the ZnO nanoparticle with the help of UV- spectroscopy (Fig. 1). The absorption spectrum of the zinc nanoparticles synthesized from *the Mentha piperata* plant specified falls at 281.5 nm, which showed the presence of ZnO. A higher absorption peak at 385 nm of ZnO nanoparticles was also observed^53^. Similar results for AgNPs were also recorded by^54^, confirming the nanoparticles with broad peaks in the 420 to 425 nm range. A slight difference in data may be due to the differences in plant species used for the green biosynthesis of ZnO NPs. In the present study, the ZnO NPs were observed in the range of 200 and 600 nm. These findings are supported by previous studies such as; synthesized Ag NPs using *Boswellia ovalifoliolata* stem bark extract fall in 200 to 800 nm^55^. According to^56^, synthesized zinc oxide nanocomposite particle occurs in the range of 200 and 400 nm, and from^57^, prepared ZnO NPs fall in the 190 to 204 nm range. These findings show a strong correlation with our current study. Furthermore, the report of^58^ indicated that 370 nm absorption of wavelength is the best range for the visibility of ZnO and ZnO hybrid nanostructures. These reports and the current results suggest that the visible appearance range of ZnO NPs falls within the 200 to 800 nm range.

### Fourier transform infrared spectroscopy (FTIR)

**Figure 2:**
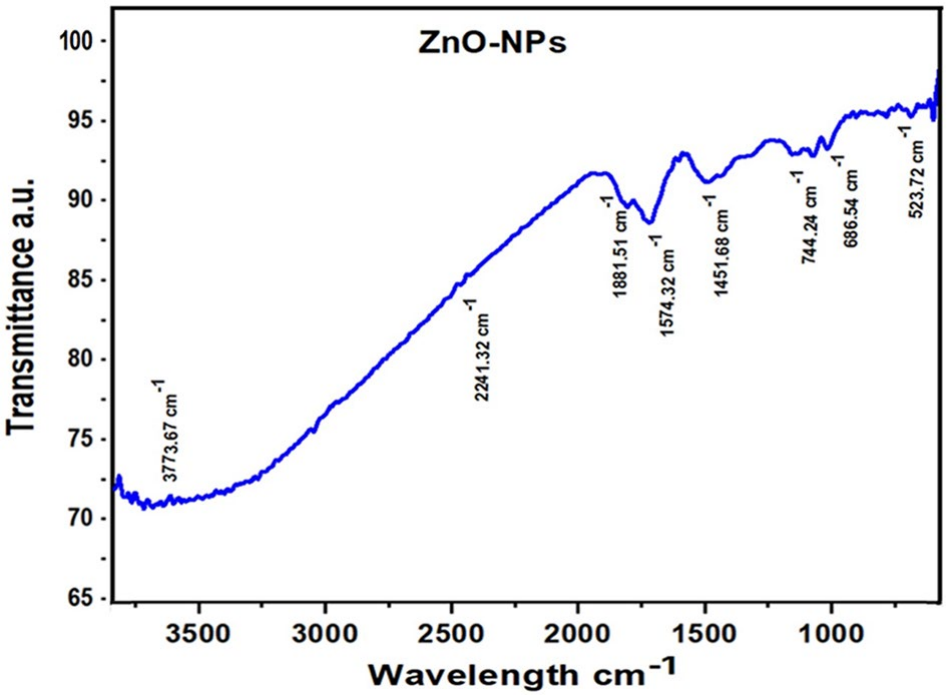
FTIR spectrum of ZnO NPs prepared from *Mentha piperita.* FTIR was performed by potassium bromide (KBr) pellet method with a settlement and examine range of 4 cm^−1^, from 400 to 4000 cm^−1^. The infrared (IR) ingestion spectra uncovered the presence of various functional groups on the particle’s surface.

**Figure 3:**
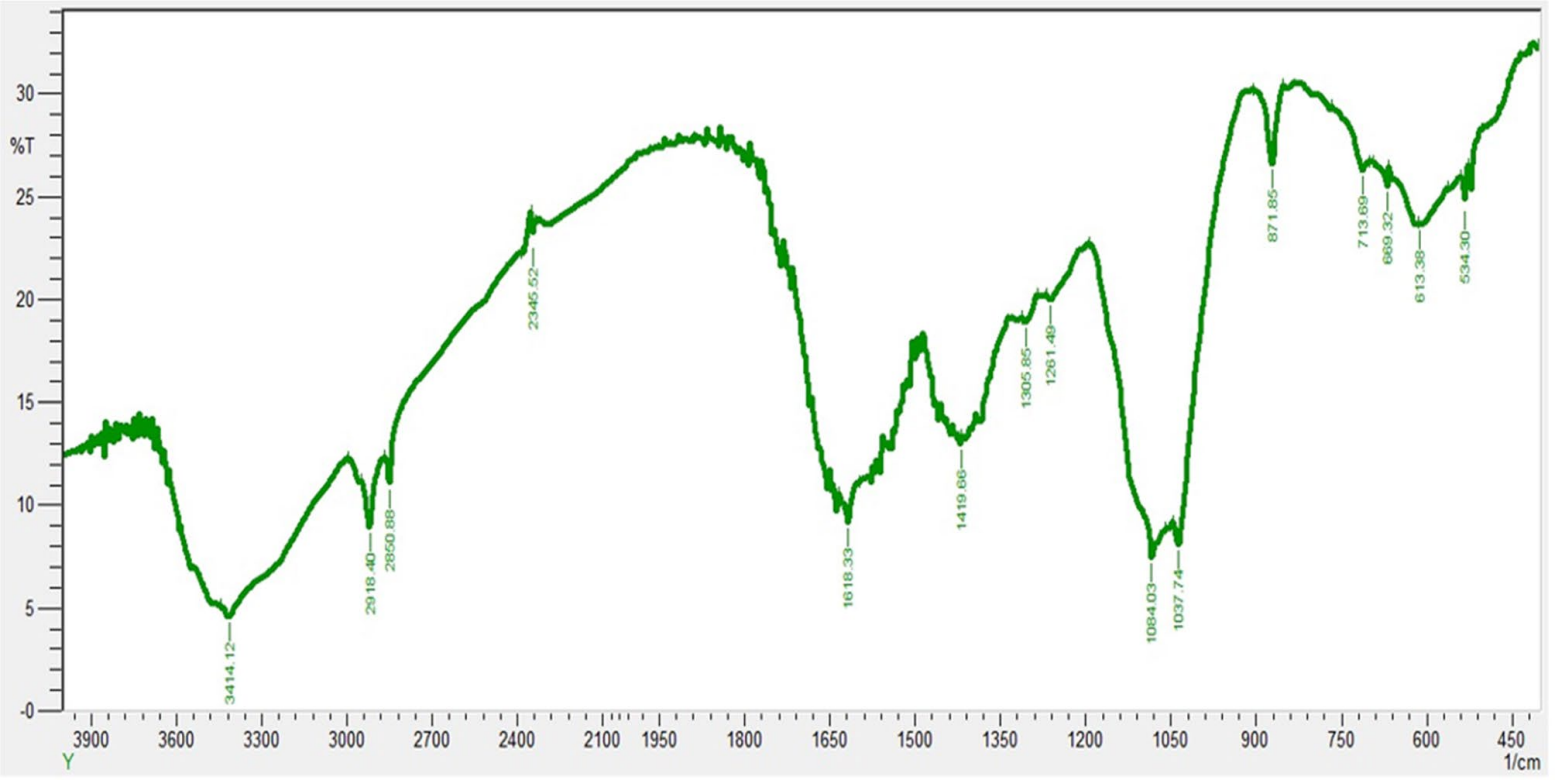
FTIR spectrum of *Mentha piperita* extract performed by potassium bromide (KBr) pellet method with a settlement and examines range of 4 cm^−1^, from 400 to 4000 cm^−1^. The infrared (IR) ingestion spectra uncovered the presence of various functional groups in the extract of *Mentha piperita*.

FTIR investigates the presence of functional groups of various phytochemicals present in the biogenic nanoparticles. The spectrums showed the range in cm^−1^ for *Mentha piperata*. FTIR analysis of ZnO NPs shows that the Carboxylic functional group (COOH) was present at 3773.67 cm^−1^. The peak at 2241.32 cm^−1^ indicates the presence of alcohol groups OH. Whereas; 1881.51 cm^−1^, 1574.32, and 1451.68 cm^−1^ specify the presence of C-H and N–H stretching. Other peaks displayed at 744.24 cm^−1^, 686.54 cm^−1^, and 523.72 cm^−1^ show numerous functional groups such as O–H, C-O of carboxylic anions and C-N nitrite respectively as shown in (Fig. 2). The FTIR spectrums for aqueous extracts of *M. piperata* showed the range in cm^−1^ with spectral wavelength between 400 and 4000 nm and investigate the presence of functional groups of various phytochemicals present in the aqueous extracts of *M. piperata*. The FTIR peaks and stretching bands commonly found in extracts, –CH (Alkyl) group at 3414.12 cm^−1^ and –CH (Alkene) group at 2918.40 cm^−1^. The wavelength at 2850.88 cm^−1^ indicated the presence of alkene (C=C) group. The wavelength 1618.33 cm^−1^ indicated the presence of Alpha–beta unsaturated ketone (C=C) group, 1419.66 for R-NO2 group, 1305.85 cm^−1^ phenol (O–H) group and 1261.49 cm^−1^ indicate Alkyl-aryl-ether (C–O) group, and 1084.03 cm^−1^ depicted the presence of aliphatic (C–O) group. The wavelength 1037.74 cm^−1^ revealed the presence of Anhydride (CO–O–CO) group and the wavelength at 871.85 cm^−1^ indicated the occupation of Alkene (C=C) group. Other peaks displayed habitation of bending at 713.69 cm^−1^, 669.32 cm^−1^, 613.38 cm^−1^, and 534.30 cm^−1^ revealed the presence of numerous functional groups such as; O–H, C–O, C–N, and C–I of carboxylic anions, nitrite and Halo compounds respectively as shown in (Fig. 3).

These results revealed that the various groups represent the presence of various polyphenolics. In contrast,^59^ reported that the FTIR results at 3314 cm^−1^ of n-type ZnO nanoparticles prepared from ginger and garlic roots represent the OH functional group. The differences in data no doubt are due to the selection of different plant extracts used for particle preparation. However, our findings are in agreement with the reported data^60^. In the present study, the peaks range from 744.24 to 686.54 cm^−1^ representing the presence of O–H, and C-O of carboxylic anions, while the findings of^61^ reported that the peaks range from 1640–3313 cm^−1^ represent phenols (O–H bond) and primary amines (C=O) in CuO NPs using the plant extract of *Syzygium alternifolium* this difference of opinion is due to the differences of plant material and salt used in the preparation of NPs. A similar result of various functional groups falling within the same range was observed by^55^ in ZnO NPs prepared from *Boswellia ovalifoliolata* stem bark. The data presented by^62^ are in agreement with the current study in which novel cafestol-loaded ZnO NPs were prepared from the diterpene in coffee (Cafestol). Ahmad et al.^63^ also observed a similar C–N group in AgNPs prepared from the leaves of *Catharanthus roseus*. The comparative evaluation of extract and biogenic ZnONPs revealed uniform functional group region which confirmed the presence of important phytochemicals such as; flavonoids, alkaloids and polyphenols.The current FTIR results suggest that these biogenic ZnO NPs of *M. piperita* are a rich source of phytochemicals.

### X-ray diffraction spectrum (XRD) Analysis

**Figure 4:**
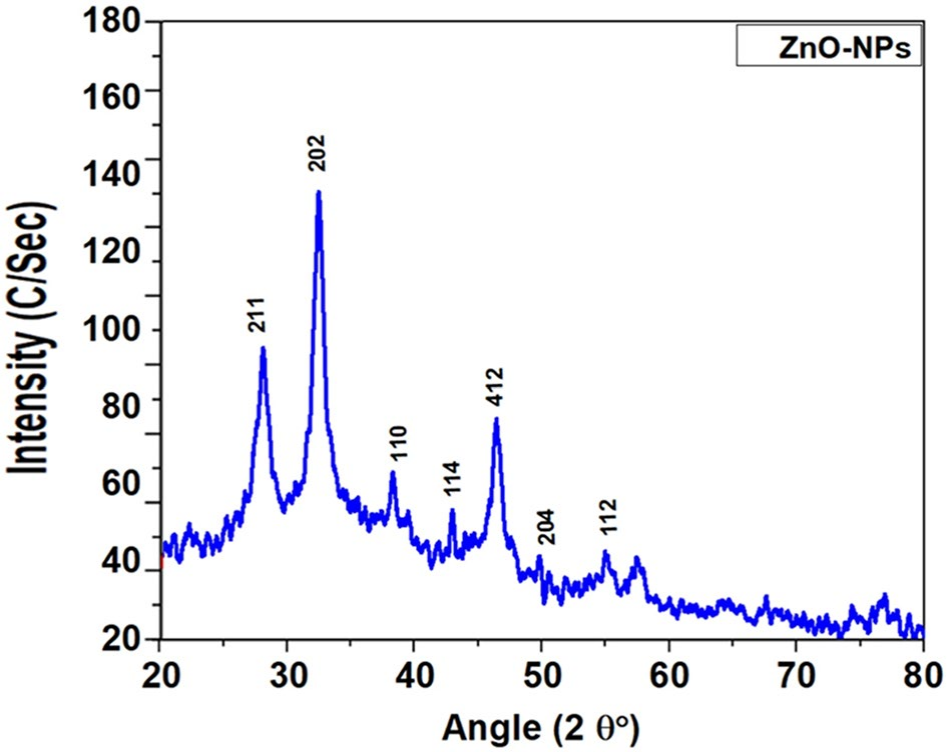
Peaks of biogenic ZnO NPs through XRD. The XRD pattern of ZnO NPs was recorded on an X-ray diffractometer at the range of 20 from 10 to 80 with a scan rate of 20/min utilizing Cu Alpha (lambda = 1.5406A) radiation with an accelerating voltage of 40 kV. Different stages present in the samples were assessed by means of X-pert high score programming having a search match office.

X-ray diffraction spectrum of biosynthesized Zinc nanoparticles from *Mentha piperata* observed Bragg peaks to XRD spectra at (211, 202, 110, 114, 412, 204, and 112). Nano grain showed characteristic peaks at 2θ Values of 28.5°, 32.3°, 39.7°, 43.6°, 46.4°, 50.2° and 55.3° that correspond to (211), (202), (110), (114), (412), (204), and (112) as shown in (Fig. 4 and Table 1) which indicated that the sample was crystalline in nature and powder size is about 22 nm at the present study. The study of Singh et al.^58^ also reported that the different ZnO phase materials displayed crystalline structures at (101) orientation at 2θ = 36.2°. The present findings exhibit a robust congruence with the documented dataset^64^. Likewise, ZnO nanoparticles synthesized from the stem bark of *Boswellia ovalifoliolata* manifested a crystalline arrangement spanning the 20° to 80° range at 2θ, and the broadening of the peaks further corroborated the nanoparticles' nanoscale size^55^, substantiating a coherent alignment with the ongoing results. The distinctive peaks located at 2θ values within the crystalline framework of ZnO nanoparticles, formulated from *M. piperata* in this study, were identified at 28.5°, 32.3°, 39.7°, 43.6°, 46.4°, 50.2°, and 55.3°. This observation displayed marked consistency with the outcomes (31.5°, 34.16°, 36°, 47.3°, 56.34°, 62.62°, 67.72°) of^65^, wherein ZnO nanoparticles were synthesized from the hairy root cultures of *Phoenix dactylifera*. Analogously,^13^ reported akin XRD results featuring diffraction peaks at 2θ values of (31.77°, 34.40°, 36.22°, 47.61°, 56.58°, 62.85°, and 67.93°), employing leaf, stem, and callus cultures of *Mussaenda frondosa*. Evidently, the findings of the current investigation and those elucidated in preceding studies collectively underscore a remarkable similarity in XRD patterns, thereby establishing a commonality in the greener synthesis of ZnO nanoparticles derived from diverse medicinal plant sources.

**Table 1:** XRD Peaks of biogenic ZnO NPs prepared from *Mentha piperata* that includes, Bragg reflection (hkl), peak position 2θ (°), FWHM Bsize (°), and DP (nm).

Bragg Reflection (hkl)	Peak position 2θ (°)	FWHM Bsize (°)	DP (nm)
211	28.5	0.357	23.34
202	32.3	0.463	47.92
110	39.7	0.482	42.27
114	43.6	0.383	33.87
412	46.4	0.512	51.36
204	50.2	0.489	48.91

### Scanning electron microscopy (SEM) and Transmission electron microscopy (TEM), Selected Area Electron Diffraction (SAED) and Zeta potential

**Figure 5:**
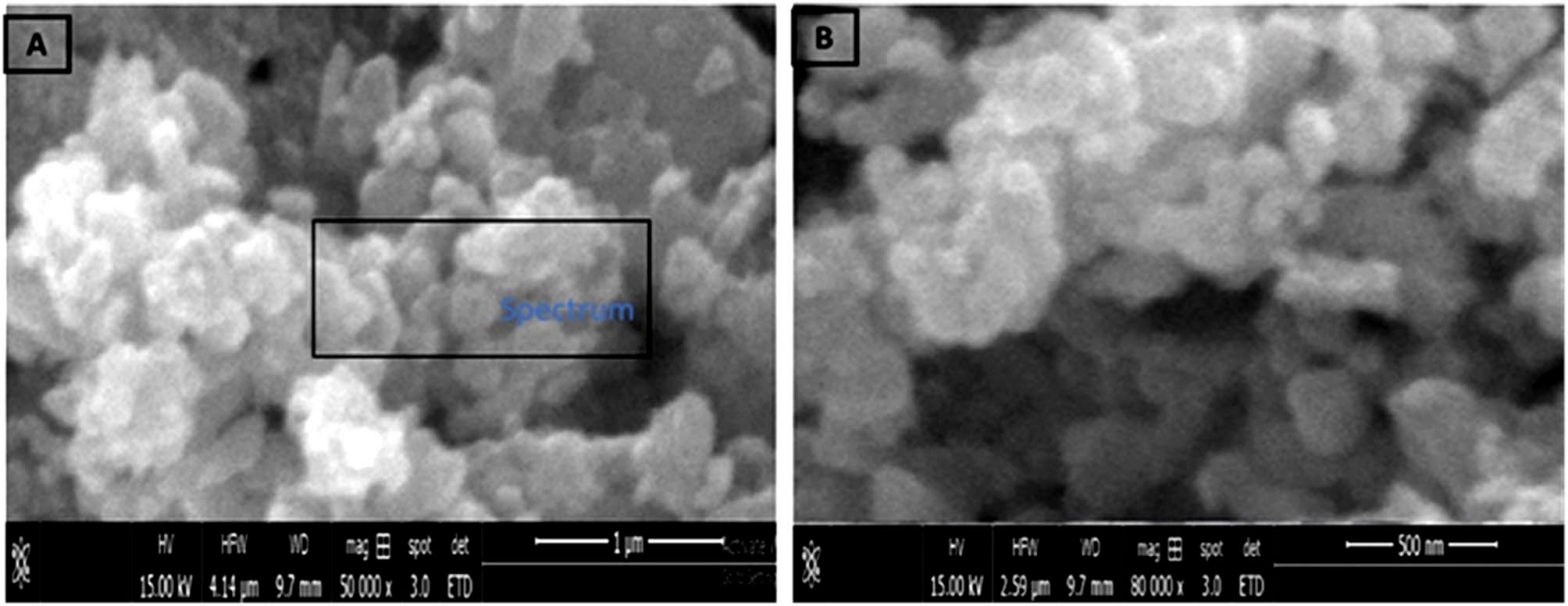
SEM of the prepared biogenic ZnO NPs. SEM (model Jeol JSM-6480LV) was utilized at 20 kV for 4 min. The ZnO NPs have pyramidal symmetry of the particle ranges 20–30 nm in size.

**Figure 6:**
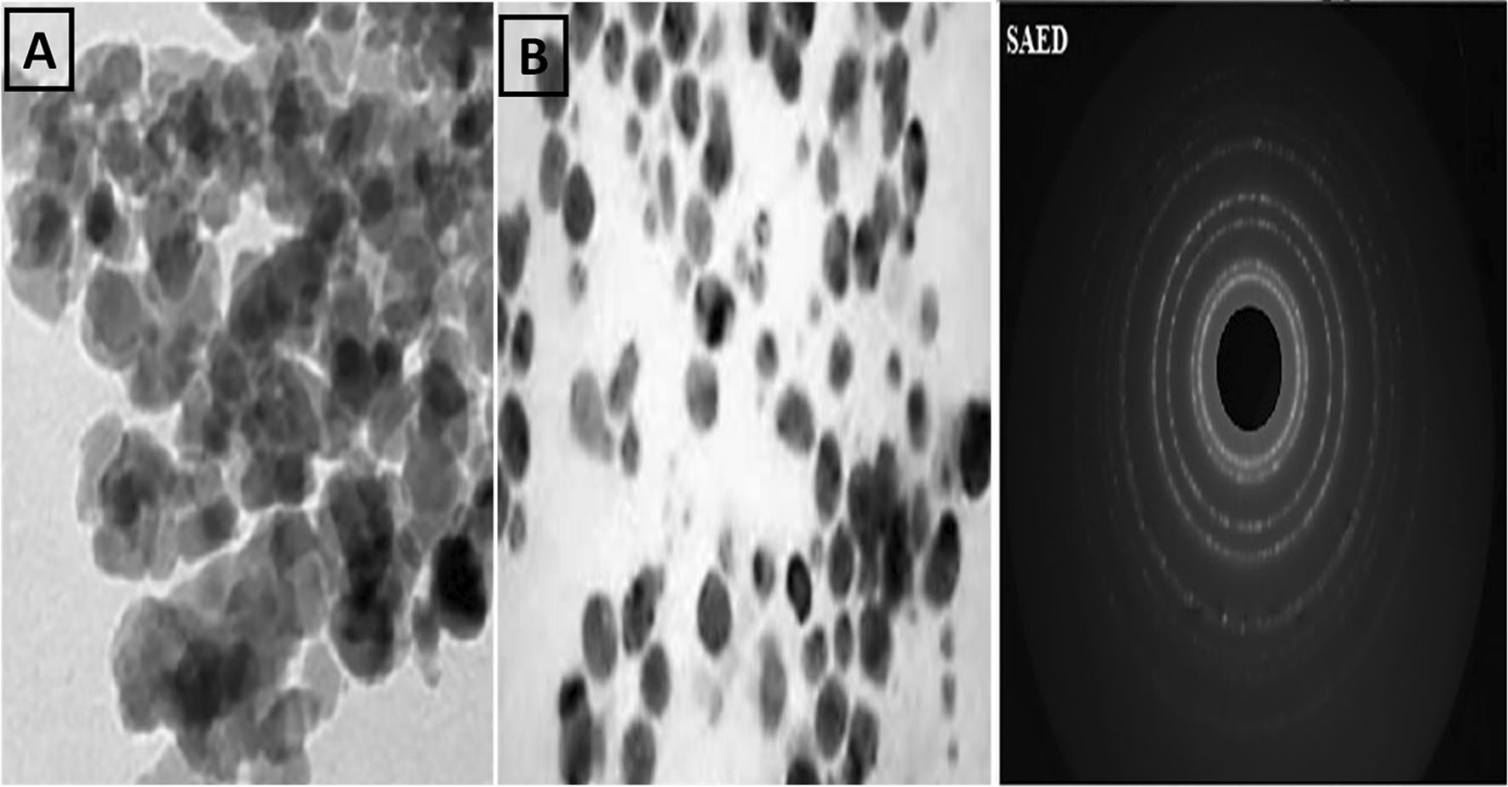
TEM micrographs along with SAED pattern of the prepared biogenic ZnO NPs. TEM (JEM-2100; manufacturer JEOL, Japan) was utilized at 200 kV. The synthesized ZnO NPs ranges 15 to 27 nm with 18 nm average in size.

**Figure 7:**
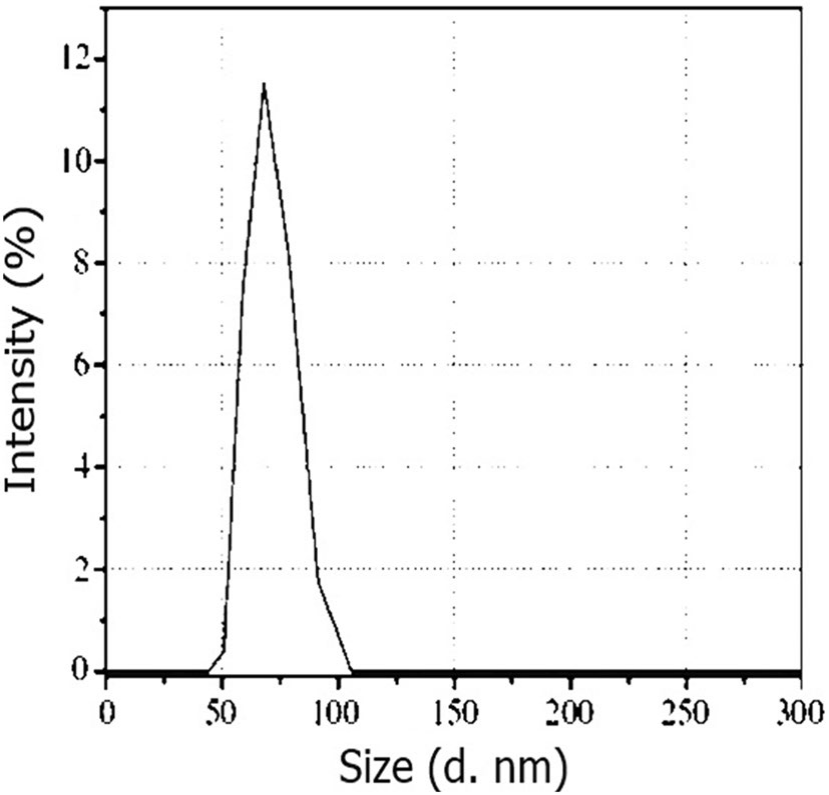
Zeta potential measurement window revealed 50 to 100 nm of the effective electric charge on the synthesized ZnONPs surface and the electro mobility. This depicts the positive charge at ZnONPs.

The SEM analysis confirmed the morphological shape of the synthesized ZnO nanoparticle. The result investigated that ZnO nanoparticles have pyramidal symmetry of particles approximately ranges between 20–30 nm (Fig. 5). The transmission electron microscopy (TEM) was carried out for determination of shape and size of synthesized ZnNPs of *Mentha piperita* extract. TEM results showed the globular and oblong shaped nanoparticles having sizes ranging from 15 to 27 nm. The mean particle size calculated was 18 nm as per TEM micrographs. The size and form of biosynthesized silver nanoparticles revealed even distribution and capping of the biomolecules present in the extract. Besides, the Brags reflection rings were observed during selected area electronic diffraction (SAED) indicating the crystalline nature of nanoparticles (Fig. 6). TEM analysis verified the efficiency of *Mentha piperita* extract for the synthesis of ZnNPs. Zeta potential identifies the charge difference between the loop of ions (which surrounds the surface of opposite charge NPs) and bulk fluid (in which NPs suspended). It is often the binding of negative charge ions with positive charge particles therefore; the stability of NPs is directly proportional in the medium to higher electrostatic repulsion. The Zeta potential window given in Fig. 7- revealed 50 to 100 nm which confirms excellent sedimentation and well dispersed nature of the synthesized NPs.

Characterization of zinc nanoparticles unveiled their spherical morphology, exhibiting a size spectrum of 7–9 nm, as investigated^66^. Correspondingly, a notably analogous outcome was documented by^62^ in the context of ZnO nanoparticles, wherein particle dimensions ranged from 45 to 60 nm, and hexagonal structure was ascertained through Zeta Sizer analysis. In congruence with these findings,^67^ reported similar outcomes, where the size of magnesium oxide nanoparticles, derived from Dalbergia sissoo extract, registered at less than 50 nm. In contrast, biogenic silver nanoparticles from Catharanthus roseus displayed a size range of 40 to 60 nm^63^. This difference in data may be due to the difference in plant material and metallic salts used for biogenic particle preparation. The current study's particle size (20 to 30 nm) was in good agreement with^65^, who reported particle size (30.87–47.89 nm) using extract of hairy root cultures of Phoenix dactylifera for ZnO NPs synthesis. Using HR-TEM images,^59^ found that the n type ZnO nanoparticles prepared from ginger and garlic roots were less than 50 nm in size. The current study's findings, as well as those reported in the literature, supported the size of ZnO NPs ranging from 20 to 60 nm. The minor differences in size and multiple symmetries are most likely due to differences in adapting different methodologies, plant species, and metallic salt selection used for nanostructure synthesis.

### EDX Analysis

**Figure 8:**
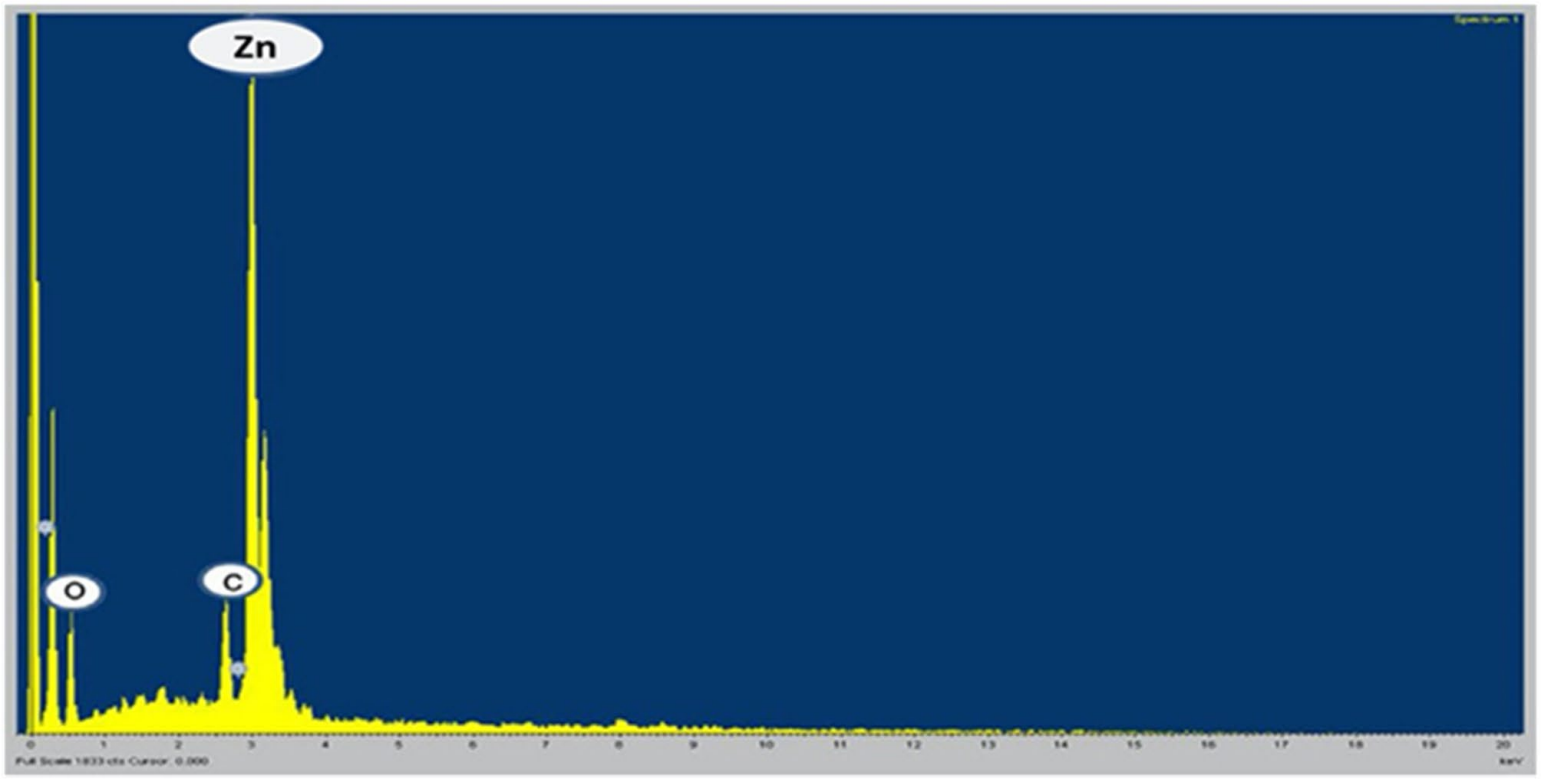
EDX analysis of Zinc Oxide nanoparticles. The specimen was resolved through EDX programming that applies ZAF correction and carries out the incorporation of the top of the plate to get the last measurement of the element in the sample.

The EDX analysis confirmed the presence of Zinc nanoparticles as spherical shaped. EDX analysis of Zinc nanoparticles revealed (Zn) Zinc, (C) Carbon, and (O) Oxygen which displayed the presence of metallic zinc acetate in the synthesis of Zinc oxide nanoparticles. EDX spectrum revealed three peaks viz., Zinc, Carbon, and Oxygen (Fig. 8), this confirmed the presence of Zinc with a strong prominent peak (85.71% weightage between the characteristic spectrum range of 3 to 4 keV).The findings of the current study are in agreement with the reported data^68^. In the present study, the strong peak of Carbon indicates that it is a capping and stabilizing agent for the formation of biogenic nanostructures of ZnO from the leaves of *M. piperita*. The work of^13^ also reported similar results while using the extracts of leaf, stem, and callus cultures of *Mussaenda frondosa* for the biogenic synthesis of ZnO NPs. The work of^56^ used the techniques of inductively coupled plasma optical emission spectroscopy for the presence of Zinc in graphene oxide/Zinc oxide and observed that the techniques demonstrated the quantity increases of Zinc in the nanocomposite. In this study, the EDX analysis confirmed the presence of Zn, C, and O in the biogenic ZnO nanostructures synthesized from *M. piperita*.

### Antimicrobial potential of Biogenic ZnO NPs

**Figure 9:**
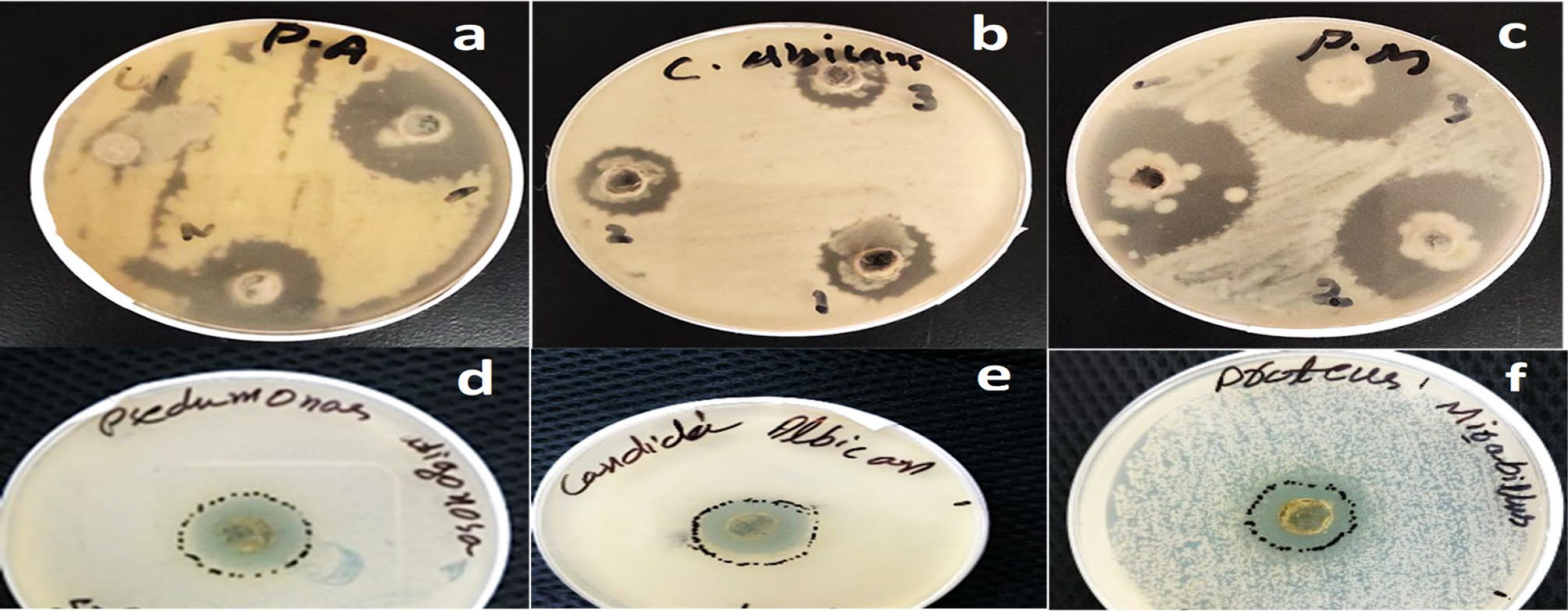
Pictorial presentation of bacterial and fungal growth inhibition by ZnO nanoparticles (**a**) effect of different concentrations of ZnO NPs against resistant *Pseudomonas aeroginosa* (**b**) *Candida albicans* (**c**) *Proteus mirabilis* (**d**) ciprofloxacin against *Pseudomonas aeruginosa* (**e**) clotrimazole against *Candida albicans* and (**f**) azithromycin against *Proteus mirabilis.*

**Figure 10:**
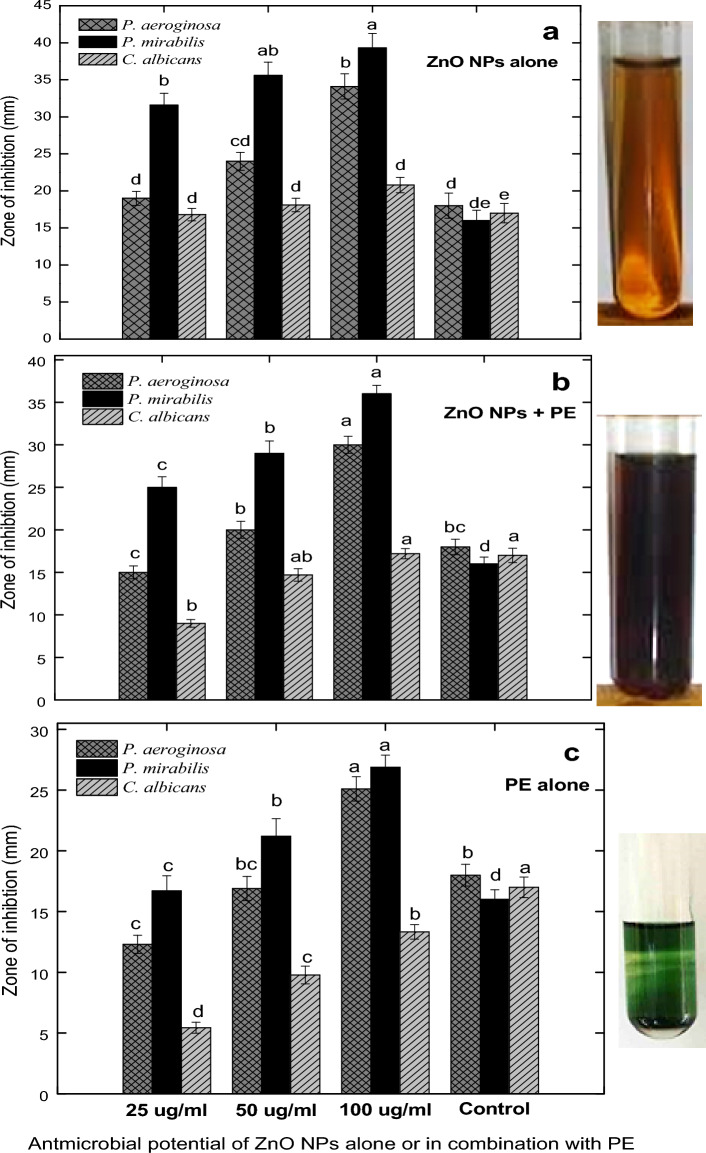
Antimicrobial potential of different concentrations (25, 50, and 100 ug/ml) of biogenic zinc oxide nanoparticles, zinc oxide nanoparticles in combination with plant extracts and plant extracts alone against pathogenic microorganisms. All the values are mean ± SE. Bars labeled with different letters (LSD values) exhibited significant variation (α < 0.05).

The Zinc oxide nanoparticles were used against UTI-resistant microbial species; *Proteus mirabilis, Pseudomonas aeruginosa*, and *Candida albicans*. Three different concentrations of ZnO nanoparticles (100, 50, and 25 ug/ml) were used against these microbes. While only the single higher concentration (100 ug/ml) of antibiotics was used. In the current study, the higher concentration (100 ug/ml) of zinc oxide NPs showed the maximum activity (34.16 ± 2.3 mm) against *Pseudomonas aeruginosa*, followed by 50 and 25 ug/ml and exhibited 24.0 ± 1.22 and 19.0 ± 3.5 mm zones of inhibitions against *Pseudomonas aeruginosa* (Figs. 9a,10a)*.* The ZnO nanoparticles were also used against *Candida albicans*. The highest concentration (100ug/mL) exhibited 20.83 ± 0.62 mm of activity while decreasing the nanoparticles concentrations, the activity also decreases (Fig. 9b). Here the highest activity (39.33 ± 3.29 mm) was observed against *Proteus mirabilis* using the higher concentration (100 ug/ml) of ZnO NPs (Fig. 9c) which was comparatively higher than *Pseudomonas aeruginosa*, *Candida albicans*, and antibiotics.

As compared to ZnO NPs alone, the combination of ZnO NPs + PE (100 ug/ml) displayed 30 ± 0.9 mm, 36 ± 1.9 mm, and 17.2 ± 0.4 mm zones of inhibitions against *Pseudomonas aeruginosa*, *Proteus mirabilis* and *Candida albicans* (Fig. 10b)*.* However, the lowest activities were displayed by applying different concentrations of PE alone (Fig. 10c). The antimicrobial potentials of ZnO NPs were compared with the antibiotics which are commonly used in UTI infections. The ciprofloxacin used against *Pseudomonas aeruginosa* displayed an 18 mm zone of inhibition (Fig. 9d). The clotrimazole exhibited a 17 mm zone of inhibition (Fig. 9e), while the azithromycin used against *Proteus mirabilis* showed a 16 mm zone of inhibition (Figs. 9f, 10). These results suggest that ZnO NPs prepared from *Mentha piperita* strongly inhibited the growth of *Proteus mirabilis, Pseudomonas aeroginosa*, and *Candida albicans*. The overall findings revealed dose-dependent activities. The findings of different studies conducted on the microbes mostly cause urinary tract infection (UTI) in humans such as^69–71^ tested the antibiotics against the authenticated strains such as; (*Pseudomonas aeruginosa* ATCC # 9721, *Candida albicans* ATCC # 14,054 and *Proteus mirabilis* ATCC # 12,453). The ciprofloxacin displayed a 45 mm zone of inhibition against *Pseudomonas aeruginosa* (ATCC # 9721), clotrimazole exhibited a 23 mm zone of inhibition against *Candida albicans* (ATCC # 14,054) and azithromycin displayed 35 mm zone of inhibition against *Proteus Mirabilis* (ATCC # 12453).

Due to the extensive use of antibiotics, the resistance in microbial cells also increases, like ciprofloxacin-resistant *P. aeruginosa* is rapidly increasing. Ciprofloxacin resistance can arise through the acquisition of mutations in genes encoding the target proteins of ciprofloxacin and regulators of efflux pumps, which leads to the over-expression of these pumps. However, nanoparticles as alternative drugs fluctuate the gene expression level and efflux pumps in such a way that cause the death of microorganism^56,62,63,67^. Nanoparticle has the ability to disturb the metabolic activity of microbial cell which is more beneficial for the treatment of various infections caused by resistant microbes and also prevent the formation of biofilm^72^. When a nanoparticle crosses the cell membrane it must affect the shape and function of the cell membrane. After that NPs must contact with fundamental constituents of microbial cells such as DNA, ribosomes, and lysosomes, inhibition of enzymes, change in gene expression, deactivation of protein, heterogeneous alternation, electrolytic disturbance, variation of cell membrane permeability, and oxidative stress^35^. Metal oxides produce metal ions that dissolved through the cell membrane which affect proteins and nucleic acid functional groups such as carboxyl (-COOH), amino (-NH), and thiol/sulfhydryl/sulfanyl group (-SH) which cause different effects on physiological mechanisms, cell structure, enzymatic activity and finally killed the microbes that is why nanotechnology are currently utilized as a source of antimicrobial agents^35,73,74^.

**Figure 11:**
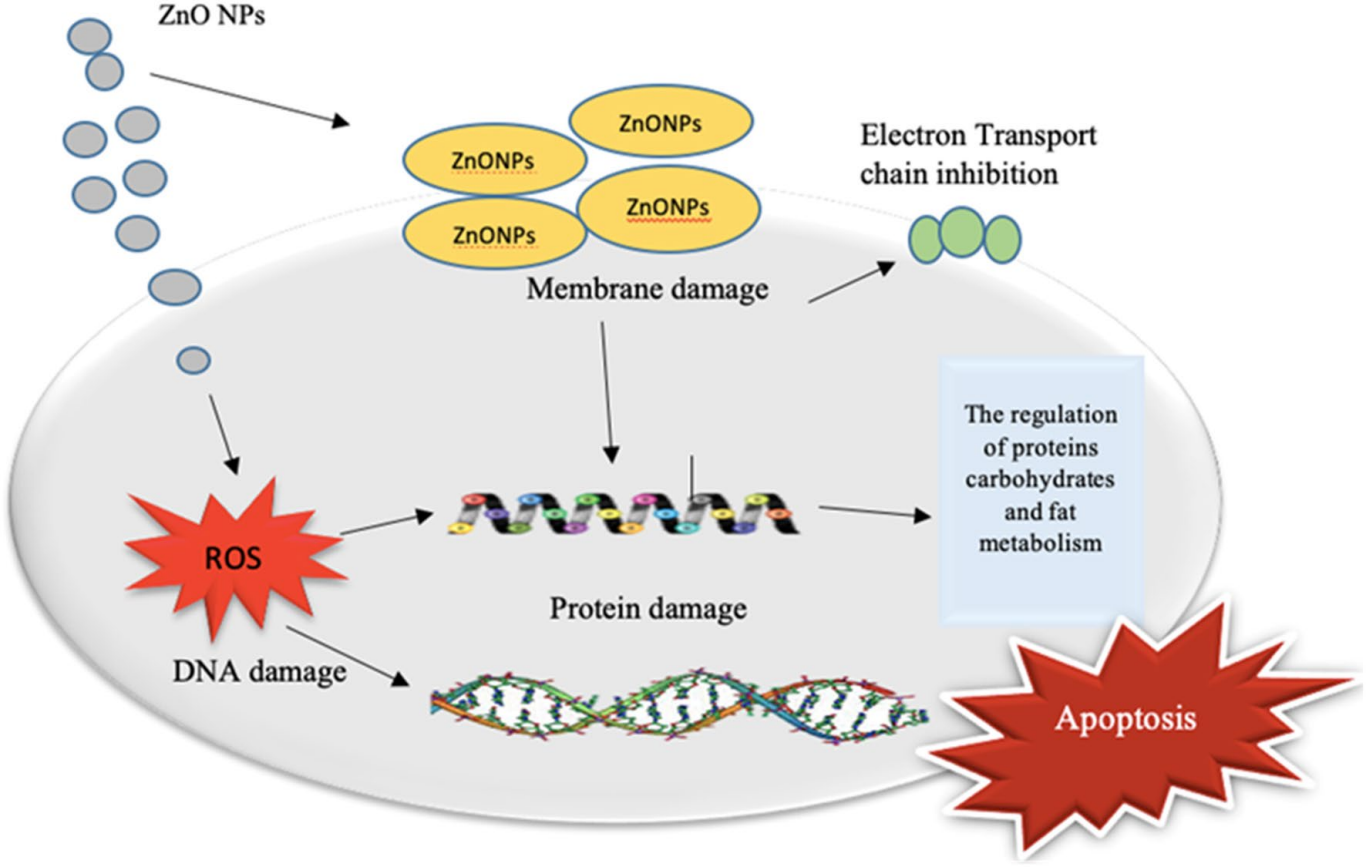
Different mechanisms of action of NPs in bacterial cells. The biogenic ZnO NPs interact with MDR microbes and activate oxidative stress mechanisms. It also inhibits enzymes and proteins as well as epigenetic changes in DNA and ultimately causes cellular leakage of the membranes leading to the death of the microorganisms.

From different sources, different microbes enter to host and cause numerous diseases^75–77^. These diseases can be controlled by various synthetic antibiotics. The frequent utilization lead to mutation and finally resistance in microorganisms^78^. Currently, multidrug-resistive strains of many microbes arises serious problems^59^. Such a problem needs alternative sources, such as the use of multiple nanoparticles prepared from medicinal plants which are natural, less toxic, and more effective against resistant pathogens. The mechanism (Fig. 11) showed how our formulated NPs get entered the cell by overcoming various barriers and how it disrupts the cell by interacting with various organelles and components of the cell. For instance: either interaction with the cell membrane may lead to distraction of the permeability of the membrane leading to cell disruption or by directly attacking the protein synthetic machinery and DNA component of the cell that may result in the loss of the cell integrity or simply to apoptosis or cell death. The aqueous extract *of Leonotis nepetifolia* flower bud was tested in different *in-vitro* activities suggesting its wide range of biomedical applications^79^. The modulated silver nanoparticles of *Cucumus sativa* (Cs-AgNPs) exhibited significant dose-dependent antimicrobial activity by agar well diffusion test against thetested pathogenic fungal and bacterial strains^80^.

## Conclusion

The phenomenon of heightened resistance exhibited by microorganisms, particularly bacteria, towards contemporary synthesized antibiotics, presents a pervasive and concerning global predicament. When subjected to adverse conditions, bacteria engage in the excretion of specific compounds or toxins as a countermeasure against the efficacy of antibiotics. Scientists endeavor to direct their investigations towards these stress-inducing molecules, devising strategies to hinder the defensive capabilities of these microorganisms. Nevertheless, the intricate landscape of bacterial mechanisms, encompassing both acquired and inherent traits, poses a substantial challenge in the meticulous design of compounds tailored to disrupt these targeted pathways. Moreover, the situations become worsened when misuse of the antibiotics particularly in case of recurring UTIs develops resistance in microorganisms. This phenomenon compels world researchers to develop cost-effective antibiotics with relatively no or few side effects. The rate of infectious diseases is increasing with the global increase in population. Therefore, the synthesis of new antimicrobial agents is the need of the day. The biogenic NPs can better fill this gap. We used *M. piperata*-derived Zinc NPs against pathogenic microbes which displayed a strong antimicrobial effect against these microbes as compared to synthetic antibiotics including ciprofloxacin, azithromycin, and clotrimazole and we further affirm that ZnO nanoparticles are more potent than medicinal plants as well as conventional and commercial drugs. As a result, we believe that Zinc Nanoparticles (NPs) derived from *M. piperata* could aid in the development of antibiotics for UTI strains that do not respond to standard treatments. However, more research is needed to determine how these naturally produced NPs work effectively.

## Data Availability

The datasets generated during and/or analyzed during the current study are available from the corresponding author on reasonable request.

## References

[1] Pokharel, B. & Karna, S. R. Antimicrobials in livestock production and its cross-domain dynamics. In *Emerging Modalities in Mitigation of Antimicrobial Resistance* 3–21 (Springer International Publishing, 2022).

[2] Rahman MRT, Fliss I, Biron E (2022). Insights in the development and uses of alternatives to antibiotic growth promoters in poultry and swine production. Antibiotics.

[3] Hemamalini N, Shanmugam SA, Kathirvelpandian A, Deepak A, Kaliyamurthi V, Suresh E (2022). A critical review on the antimicrobial resistance, antibiotic residue and metagenomics-assisted antimicrobial resistance gene detection in freshwater aquaculture environment. Aquac. Res..

[4] Wareth G, Dadar M, Ali H, Hamdy ME, Al-Talhy AM, Elkharsawi AR, Neubauer H (2022). The perspective of antibiotic therapeutic challenges of brucellosis in the Middle East and North African countries: Current situation and therapeutic management. Transbound. Emerg. Dis..

[5] Rose, W., Volk, C., Dilworth, T. J., & Sakoulas, G. Approaching 65 years: Is it time to consider retirement of vancomycin for treating methicillin-resistant *Staphylococcus aureus* endovascular infections? In *Open Forum Infectious Diseases* (Vol. 9, No. 5, p. ofac137). Oxford University Press (2022). 10.1093/ofid/ofac137 PMC9043000 35493116

[6] Vercelli C, Gambino G, Amadori M, Re G (2022). Implications of Veterinary Medicine in the comprehension and stewardship of antimicrobial resistance phenomenon. From the origin till nowadays. Vet. Anim. Sci..

[7] Li G, Walker MJ, De Oliveira DM (2022). Vancomycin resistance in enterococcus and *Staphylococcus aureus*. Microorganisms.

[8] Falkow S, Kennedy D (2005). Antibiotics, animals, and people–again!. Science.

[9] Walsh C (2003). Where will new antibiotics come from?. Nat. Rev. Microbiol..

[10] Ghimire J, Guha S, Nelson BJ, Morici LA, Wimley WC (2022). The remarkable innate resistance of burkholderia bacteria to cationic antimicrobial peptides: Insights into the mechanism of AMP resistance. J. Membr. Biol..

[11] Rodriguez E, McDaniel R (2001). Combinatorial biosynthesis of antimicrobials and other natural products. Curr. Opin. Microbiol..

[12] Wareth G, Dadar M, Ali H, Hamdy ME, Al-Talhy AM, Elkharsawi AR, El Tawab AAA, Neubauer H (2022). The perspective of antibiotic therapeutic challenges of brucellosis in the Middle East and North African countries: Current situation and therapeutic management. Transbound. Emerg. Dis..

[13] Jayappa MD, Ramaiah CK, Kumar MA, Suresh D, Prabhu A, Devasya RP, Sheikh S (2020). Green synthesis of zinc oxide nanoparticles from the leaf, stem and in vitro grown callus of *Mussaendafrondosa* L.: characterization and their applications. Appl. Nanosci..

[14] Rahman SU, Ullah Z, Ali A, Aziz MA, Alam N, Sher H, Ali I (2022). Traditional knowledge of medicinal flora among tribal communities of Buner Pakistan. Phytomed. Plus.

[15] Sher H, Ali A, Ullah Z, Sher H (2022). Alleviation of poverty through sustainable management and market promotion of medicinal and aromatic plants in Swat. Pakistan. Ethnobot. Res. Appl..

[16] Ullah Z, Ali U, Ali S, Ali A, Alam N, Sher H (2021). Medicinal flora and cultural values of Arkot-Biakand Valley Hindu Kush Region Swat, Pakistan.

[17] Gurib-Fakim A (2006). Medicinal plants: Traditions of yesterday and drugs of tomorrow. Mol. Aspects Med..

[18] Dickson M, Gagnon JP (2004). Key factors in the rising cost of new drug discovery and development. Nat. Rev. Drug Discov..

[19] McKay DL, Blumberg JB (2006). A review of the bioactivity and potential health benefits of chamomile tea (*Matricariarecutita* L.). Phytother. Res..

[20] Punit PS, Mello PMD (2012). A review of medicinal uses and pharmacological effects of *Menthapiperita*. Nat. Prod. Rad..

[21] Baliga MS, Rao S (2010). Radioprotective potential of mint: a brief review. J. Cancer Res. Therap..

[22] Mehta J, Naruka R, Sain M, Dwivedi A, Sharma D, Mirza J (2012). An efficient protocol for clonal micropropagation of *Menthapiperita* L. (Pipperment). Asian J. Plant Sci. Res..

[23] Sumathi S, Tharmaraj P, Sheela CD, Ebenezer R, SaravanaBhava P (2011). Synthesis, characterization, NLO study, and antimicrobial activities of metal complexes derived from 3-(3-(2-hydroxyphenyl)-3-oxoprop-1-enyl)-4H-chromen-4-one and sulfanilamide. J. Coord. Chem..

[24] Kamatou GP, Vermaak I, Viljoen AM, Lawrence BM (2013). Menthol: a simple monoterpene with remarkable biological properties. Phytochemistry.

[25] Bupesh G, Amutha C, Nandagopal S, Ganeshkumar A, Sureshkumar P, Murali K (2007). Antibacterial activity of *Menthapiperita* L. (peppermint) from leaf extracts-a medicinal plant. Acta Agric. Slov..

[26] Jaggard KW, Qi A, Ober ES (2010). Possible changes to arable crop yields by 2050. Philos. Trans. R. Soc. Lond. B Biol. Sci..

[27] Pramanik P, Krishnan P, Maity A, Mridha N, Mukherjee A, Rai V (2020). Application of nanotechnology in agriculture. Environ. Nanotechnol..

[28] Nikoobakht B, El-Sayed MA (2003). Preparation and growth mechanism of gold nanorods (NRs) using seed-mediated growth method. Chem Mater..

[29] Pontes JF, Grenha A (2020). Multifunctional nanocarriers for lung drug delivery. Nanomaterials.

[30] Kaur P, Mishra V, Shunmugaperumal T, Goyal AK, Ghosh G, Rath G (2020). Inhalable spray dried lipidnanoparticles for the co-delivery of paclitaxel and doxorubicin in lung cancer. J. Drug Deliv. Sci. Technol..

[31] Velino C, Carella F, Adamiano A, Sanguinetti M, Vitali A, Catalucci D, Bugli F, Iafisco M (2019). Nanomedicine approaches for the pulmonary treatment of cystic fibrosis. Front. Bioeng. Biotechnol..

[32] Gupta, A. K., Singh, A. & Singh, S. Diagnosis of tuberculosis: nanodiagnostics approaches. In *NanoBio. Med* (261–283). Springer (2020).

[33] Dhayanandamoorthy Y, Antoniraj MG, Kandregula CA, Kandasamy R (2020). Aerosolized hyaluronic acid decorated, ferulic acid loaded chitosan nanoparticle: A promising asthma control strategy. Int. J. Pharma..

[34] Xu Y, Liu H, Song L (2020). Novel drug delivery systems targeting oxidative stress in chronic obstructive pulmonary disease: a review. J. Nanobiotechnol..

[35] Wang L, Hu C, Shao L (2017). The antimicrobial activity of nanoparticles: present situation and prospects for the future’. Int. J. Nanomed..

[36] Kumar SS, Venkateswarlu P, Rao VR, Rao GN (2013). Synthesis, characterization and optical properties of zinc oxide nanoparticles. Int. Nano Lett..

[37] Kessler R (2011). Engineered nanoparticles in consumer products: Understanding a new ingredient. Environ. Health Perspect..

[38] Vafaee M, Ghamsari MS (2007). Preparation and characterization of ZnO nanoparticles by a novel sol–gel route. Mat. Lett..

[39] Gnanasangeetha D, SaralaThambavani D (2013). One pot synthesis of zinc oxide nanoparticles via chemical and green method. Res. J. Mat. Sci..

[40] Raghupathi KR, Koodali RT, Manna AC (2011). Size-dependent bacterial growth inhibition and mechanism of antibacterial activity of zinc oxide nanoparticles. Langmuir.

[41] Padmavathy N, Vijayaraghavan R (2008). Enhanced bioactivity of ZnO nanoparticles—an antimicrobial study. Sci. Technol. Adv. Mate..

[42] Kotloff KL, Winickoff JP, Ivanoff B, Clemens JD, Swerdlow DL, Sansonetti PJ, Adak GK, Levine MM (1999). Global burden of Shigella infections: implications for vaccine development and implementation of control strategies. Bull. World Health Org..

[43] Fan Z, Lu JG (2005). Zinc oxide nanostructures: Synthesis and properties. J. Nanosci. Nanotechnol..

[44] Song Z, Kelf TA, Sanchez WH, Roberts MS, Rička J, Frenz M, Zvyagin AV (2011). Characterization of optical properties of ZnO nanoparticles for quantitative imaging of transdermal transport. Biomed. Optic Exp..

[45] Gemmill JD, Lifson WK, Rae AP, Hillis WS, Dunn FG (1993). Assessment by general practitioners of suitability of thrombolysis in patients with suspected acute myocardial infarction. Heart.

[46] Fazal HI, Ahmad N, Ullah I, Inayat H, Khan L, Abbasi BH (2011). Antibacterial potential in *Partheniumhysterophorus*, *Stevia rebaudiana* and *Ginkgo biloba*. Pak. J. Bot..

[47] Islam AM, Khalik MF, Uddin N, Hossain MS, Hossain MM, Hasan MM, Fahad SM, Saha P (2015). Evaluation of microbiological and physicochemical profile of some herbal preparations manufactured by pharmaceutical and herbal manufacturers in Bangladesh. J. Pharm. Investig..

[48] Shaikh NM, Patel AA, Mehta SA, Patel ND (2013). Isolation and screening of cellulolytic bacteria inhabiting different environment and optimization of cellulase production. Univ. J. Environ. Res. Technol..

[49] Hassan MM, Amin KB, Ahaduzzaman M, Alam M, Faruk MS, Uddin I (2014). Antimicrobial resistance pattern against *E. coli* and Salmonella in layer poultry. Res. J. Vet. Pract..

[50] Badshah I, Mustafa N, Khan R, Mashwani ZUR, Raja NI, Almutairi MH, Sawati L (2023). Biogenic titanium dioxide nanoparticles ameliorate the effect of salinity stress in wheat crop. Agronomy.

[51] Ahmad M, Ali A, Ullah Z, Sher H, Dai D-Q, Ali M, Iqbal J, Zahoor M, Ali I (2022). Biosynthesized silver nanoparticles using *Polygonatum germiniflorum* efficiently controls *Fusarium* wilt disease of tomato. Front. Bioeng. Biotechnol..

[52] Fazal H, Ahmad N, Abbasi BH, Abbass N (2012). Selected medicinal plants used in herbal industries; their toxicity against pathogenic microoraganisms. Pak. J. Bot..

[53] Al-Hussain AJ, Abd FG, Alkaim AF, Al-Azzawi A (2017). Eco friendly synthesis, characterization and antibacterial activity of ZnO nanoparticles using *Bacillus subtilis* against multi-drug resistant bacteria. J. Global Pharma. Technol..

[54] Chauhan R, Reddy A, Abraham J (2015). Biosynthesis of silver and zinc oxide nanoparticles using *Pichiafermentans* JA2 and their antimicrobial property. Appl. Nanosci..

[55] Supraja N, Prasad TN, Krishna TG, David E (2016). Synthesis, characterization, and evaluation of the antimicrobial efficacy of *Boswellia ovalifoliolata* stem bark-extract-mediated zinc oxide nanoparticles. Appl. Nanosci..

[56] Prema D, Prakash J, Vignesh S, Veluchamy P, Ramachandran C, Samal DB, Oh DH, Sahabudeen S, Venkatasubbu GD (2020). Mechanism of inhibition of graphene oxide/zinc oxide nanocomposite against wound infection causing pathogens. Appl. Nanosci..

[57] Fakhroueian Z, Katouzian F, Esmaeilzadeh P, Bidhendi SM, Esmaeilzadeh P (2019). Enhanced engineered ZnO nanostructures and their antibacterial activity against urinary, gastrointestinal, respiratory and dermal genital infections. Appl. Nanosci..

[58] Singh V, Dwivedi LM, Baranwal K, Asthana S, Sundaram S (2018). Oxidized guar gum–ZnO hybrid nanostructures: synthesis, characterization and antibacterial activity. Appl. Nanosci..

[59] Ali M, Ikram M, Ijaz M, Ul-Hamid A, Avais M, Anjum AA (2020). Green synthesis and evaluation of n-type ZnO nanoparticles doped with plant extract for use as alternative antibacterials. Appl. Nanosci..

[60] Dhanemozhi AC, Rajeswari V, Sathyajothi S (2017). Green synthesis of zinc oxide nanoparticle using green tea leaf extract for supercapacitor application. Mater Today Proc..

[61] Yugandhar P, Vasavi T, Devi PU, Savithramma N (2017). Bioinspired green synthesis of copper oxide nanoparticles from *Syzygiumalternifolium* (Wt.) Walp: Characterization and evaluation of its synergistic antimicrobial and anticancer activity. Appl. Nanosci..

[62] Ballıca G, Çevikbaş H, Ulusoy S, Yıldırım Y (2020). The synthesis of novel cafestol loaded zinc oxide nanoparticles and their characterization. Appl. Nanosci..

[63] Ahmad S, Tauseef I, Haleem KS, Khan K, Shahzad M, Ali M, Sultan F (2020). Synthesis of silver nanoparticles using leaves of *Catharanthusroseus* and their antimicrobial activity. Appl. Nanosci..

[64] Dinesh VP, Biji P, Anuradha A, Dhara SK, Kamaruddin M, Tyagi AK, Raj B (2014). Plasmon-mediated highly enhanced photocatalytic degradation of industrial textile efuent dyes using hybrid ZnO@ Ag core-shell nanorod. RSC Adv..

[65] Naser R, Abu-Huwaij R, Al-khateeb I, Abbas MM, Atoom AM (2021). Green synthesis of zinc oxide nanoparticles using the root hair extract of *Phoenix dactylifera*: antimicrobial and anticancer activity. Appl. Nanosci..

[66] Kumar PMA, Suresh D, Nagabhushana H, Sharma SC (2015). *Beta vulgaris* aided green synthesis of ZnO nanoparticles and their luminescence, photocatalytic and antioxidant properties. Eur. Phys. J. Plus.

[67] Khan MI, Akhtar MN, Ashraf N, Najeeb J, Munir H, Awan TI, Tahir MB, Kabli MR (2020). Green synthesis of magnesium oxide nanoparticles using *Dalbergiasissoo* extract for photocatalytic activity and antibacterial efficacy. Appl. Nanosci..

[68] Bala N, Saha S, Chakraborty M, Maiti M, Das S, Basu R, Nandy P (2015). Green synthesis of zinc oxide nanoparticles using *Hibiscussubdarifa* leaf extract: efect of temperature on synthesis, anti-bacterial activity and anti-diabetic activity. RSC Adv..

[69] Thakur, M., Poojary, S. & Swain, N. Green synthesis of iron oxide nanoparticles and its biomedical applications. *Nanotechnol. Appl. Health Environ. Sci.* 83–109 (2021).

[70] Kline KA, Schwartz DJ, Lewis WG, Hultgren SJ, Lewis AL (2011). Immune activation and suppression by group *B streptococcus* in a murine model of urinary tract infection. Infect Immun..

[71] Ronald A (2002). The etiology of urinary tract infection: Traditional and emerging pathogens. Am. J. Med..

[72] Kulshrestha S, Qayyum S, Khan AU (2017). Antibiofilm efficacy of green synthesized graphene oxide-silver nanocomposite using *Lagerstroemia speciosa* floral extract: a comparative study on inhibition of gram-positive and gram-negative biofilms. Micro Pathogen.

[73] Arif M, Ullah R, Ahmad M, Ali A, Ullah Z, Ali M, Sher H (2022). Green synthesis of silver nanoparticles using *Euphorbiawallichii* leaf extract: Its antibacterial action against citrus canker causal agent and antioxidant potential. Molecules.

[74] Khan S, Bibi G, Dilbar S, Iqbal A, Ahmad M, Ali A, Ullah Z, Jaremko M, Iqbal J, Ali M (2022). Biosynthesis and characterization of iron oxide nanoparticles from *Menthaspicata* and screening its combating potential against *Phytophthorainfestans*. Front. Plant Sci..

[75] Tariq A, Shah GM, Zada A, Ali A, Shah AZ, Fatima I (2021). Phytochemical analysis and in-vitro anti-bacterial and anti-fungal activity of *Verbascumarianthum* (Benth). Pure Appl. Biol..

[76] Aslam B, Wang W, Arshad MI, Khurshid M, Muzammil S, Rasool MH, Nisar MA, Alvi RF, Aslam MA, Qamar MU, Salamat MK (2018). Antibiotic resistance: a rundown of a global crisis. Infect. Drug Resist..

[77] Dilbar S, Sher H, Binjawhar DN, Ali A, Ali IA (2023). Novel based synthesis of silver/silver chloride nanoparticles from *Stachysemodi* efficiently controls *Erwiniacarotovora*, the causal agent of blackleg and soft rot of potato. Molecules.

[78] Wang W, Hao Y, Liu Y, Li R, Huang DB, Pan YY (2021). Nanomedicine in lung cancer: Current states of overcoming drug resistance and improving cancer immunotherapy. Nanomed. Nanobiotechnol..

[79] Nagaraja SK, Niazi SK, Bepari A, Assiri RA, Nayaka S (2022). Leonotis nepetifolia flower bud extract mediated green synthesis of silver nanoparticles, their characterization, and in vitro evaluation of biological applications. Materials.

[80] Nagaraja SK, Kumar RS, Chakraborty B, Hiremath H, Almansour AI, Perumal K, Gunagambhire PV, Nayaka S (2023). Biomimetic synthesis of silver nanoparticles using Cucumis sativus var. hardwickii fruit extract and their characterizations, anticancer potential and apoptosis studies against Pa-1 (Human ovarian teratocarcinoma) cell line via flow cytometry. Appl. Nanosci..

